# Mitochondrial genome assembly of the Chinese endemic species of *Camellia luteoflora* and revealing its repetitive sequence mediated recombination, codon preferences and MTPTs

**DOI:** 10.1186/s12870-025-06461-6

**Published:** 2025-04-05

**Authors:** Xu Xiao, Zhaohui Ran, Chao Yan, Weihao Gu, Zhi Li

**Affiliations:** College of Forestry, Guizhou University, Guiyang, 550025 China

**Keywords:** *Camellia luteoflora*, Mitochondrial genome, Homologous recombination, RNA editing events

## Abstract

*Camellia luteoflora* Y.K. Li ex Hung T. Chang & F.A. Zeng belongs to the *Camellia* L. genus (Theaceae Mirb.). As an endemic, rare, and critically endangered species in China, it holds significant ornamental and economic value, garnering global attention due to its ecological rarity. Despite its conservation importance, genomic investigations on this species remain limited, particularly in organelle genomics, hindering progress in phylogenetic classification and population identification. In this study, we employed high-throughput sequencing to assemble the first complete mitochondrial genome of *C. luteoflora* and reannotated its chloroplast genome. Through integrated bioinformatics analyses, we systematically characterized the mitochondrial genome’s structural organization, gene content, interorganellar DNA transfer, sequence variation, and evolutionary relationships.Key findings revealed a circular mitochondrial genome spanning 587,847 bp with a GC content of 44.63%. The genome harbors70 unique functional genes, including 40 protein-coding genes (PCGs), 27 tRNA genes, and 3 rRNA genes. Notably, 9 PCGs contained 22 intronic regions. Codon usage analysis demonstrated a pronounced A/U bias in synonymous codon selection. Structural features included 506 dispersed repeats and 240 simple sequence repeats. Comparative genomics identified 19 chloroplast-derived transfer events, contributing 29,534 bp (3.77% of total mitochondrial DNA). RNA editing prediction revealed 539 C-to-T conversion events across PCGs. Phylogenetic reconstruction using mitochondrial PCGs positioned *C. luteoflora* in closest evolutionary proximity to *Camellia sinensis* var. *sinensis*. Selection pressure analysis (Ka/Ks ratios < 1 for 11 PCGs) and nucleotide diversity assessment (Pi values: 0–0.00711) indicated strong purifying selection and low sequence divergence.This study provides the first comprehensive mitochondrial genomic resource for *C. luteoflora*, offering critical insights for germplasm conservation, comparative organelle genomics, phylogenetic resolution, and evolutionary adaptation studies in *Camellia* species.

**Supplementary Information:**

The online version contains supplementary material available at 10.1186/s12870-025-06461-6.

## Introduction

*Camellia luteoflora* Y.K. Li ex Hung T. Chang & F.A. Zeng, a perennial evergreen shrub or small tree within the *Camellia* L. genus (Theaceae Mirb.), was first discovered in Chishui City, Guizhou Province, China, in November 1981. Prof. Chang Hongda formally described it as a novel species, designating the taxonomic classification “*Camellia* L.– sect. *Luteoflora* Chang” [1]. This species exhibits a highly restricted distribution, primarily limited to the Jinsha Gou and Sidong Gou valleys in Chishui, Guizhou [2, 3], with sporadic occurrences reported in adjacent regions such as Guihua Town, Gulin County, Sichuan [4].Recognized for its ecological and botanical significance, *C. luteoflora* was designated as a nationally protected plant in China in 1983, with strict prohibitions on wild harvesting or transplantation [5]. Subsequent conservation efforts included the establishment of the Chishui Alsophila spinulosa Nature Reserve in 1984, which incorporated the species’ native habitat into its protected zones and prioritized its preservation [6]. By 1988, it was classified as a Grade I rare and endangered plant in Guizhou Province [6]. The 2013 Red List of China’s Biodiversity– Higher Plants Volume further assessed its conservation status as Vulnerable (VN), emphasizing its endemic status and escalating threats [7]. Currently, anthropogenic pressures, including habitat degradation and fragmentation, have precipitated a severe population decline in *C. luteoflora*. Both population size and individual numbers are diminishing rapidly, pushing this taxon toward critical endangerment. Urgent interdisciplinary conservation strategies integrating genomic insights, habitat restoration, and policy enforcement are imperative to mitigate its extinction risk.

Recent years have witnessed growing scientific interest in *C. luteoflora*, with studies spanning multiple aspects of its biology and ecology. Zhang Ting investigated the species’ asexual propagation potential, demonstrating that hormone treatments significantly influence rooting efficiency in cuttings derived from wild germplasm within its natural habitat [8]. Concurrently, Liu Haiyan identified optimal seed germination conditions through systematic propagation experiments, advancing cultivation protocols for this species [9]. Further contributions by Zou Tiancai et al. integrated comparative trait polarity analysis, biogeographical approaches, and phylogenetic evolutionary frameworks to elucidate the origin, distribution patterns, and adaptive features of *C. luteoflora*, complemented by investigations into its leaf anatomy and photosynthetic characteristics [10, 11].While current research has prioritized unraveling the species’ evolutionary origins, refining breeding techniques, mapping spatial distributions, and diagnosing endangerment drivers [12, 13], critical gaps remain in genetic studies. Notably, comprehensive genomic analyses of its mitochondrial and chloroplast genomeskey resources for resolving phylogenetic relationships and genetic diversityare still lacking.

Mitochondria, the primary energy-producing organelles in eukaryotic cells, drive aerobic respiration and play multifaceted roles in regulating critical metabolic processes such as cell differentiation, apoptosis, proliferation, and stress response [14]. Beyond their metabolic functions, they are intrinsically linked to plant growth vigor and cytoplasmic male sterility (CMS), traits of significant agricultural and evolutionary importance [15]. These features position mitochondrial studies as vital tools for investigating eukaryotic evolution, species identification, genetic diversity, and molecular breeding strategies [16]. The mitochondrial genome exhibits distinctive characteristics: while relatively compact in size with a conserved gene repertoire and dense gene arrangement, it also contains hypervariable noncoding regions that contribute to genomic diversity [17]. This contrasts with chloroplast genomes in higher plants, which display minimal homologous recombination and maintain strict conservation in gene number, order, and composition [18]. Notably, mitochondrial genomes balance evolutionary conservation with unique divergence patterns, evolving at rates distinct from nuclear genes. Their relatively large size and structural complexity provide rich taxonomic information, enabling resolution of classification challenges among closely related species [19, 20].

Plant mitochondrial DNA (mtDNA) exhibits remarkable structural complexity, characterized by dynamic configurations including primary circular molecules, subgenomic circular forms, linear arrangements, and highly branched multigenomic architectures [21, 22]. For instance, mitochondrial genome assembly in Salvia miltiorrhiza Bunge revealed two distinct unit maps reflecting its branched multigenomic organization [23, 24]. This structural diversity is further amplified by abundant repetitive sequences within plant mtDNA, which drive frequent homologous recombination events [25, 26]. Consequently, mitochondrial genomes may exist as singular or multiple circular/linear DNA conformations, often coexisting within a single organism [27]. To explain this plasticity, researchers have proposed models such as the “master circle” hypothesis and the multichromosome framework. The former posits a dynamic multipartite system, where a primary “master circle” containing the full genomic content interconverts with smaller subgenomic circles via recombination at repetitive regions [28]. Advances in hybrid sequencing strategies (combining second- and third-generation technologies) have now enabled precise resolution of these intricate multipartite structures [29].

In this study, we present the first complete assembly and annotation of both mitochondrial and chloroplast genomes for *C. luteoflora*. Through comparative analyses, we characterized fundamental genomic architecture, structural conformations, GC content, codon usage bias, repetitive elements, RNA editing sites, and evolutionary selection pressures (Ka/Ks ratios) in its mitochondrial genome. Furthermore, we systematically investigated interorganellar sequence transfers between mitochondrial and chloroplast genomes, providing critical insights into DNA exchange mechanisms and functional interdependencies between these organelles. These findings advance understanding of plant mitochondrial genome organization—particularly repeat-mediated recombination dynamics—while establishing a foundation for exploring organellar genome coevolution and horizontal gene transfer within the *Camellia* genus.

## Materials and methods

### Collection of plant material, DNA extraction and its sequencing

*C. luteoflora* specimens were collected from their natural habitat in Jinshagou, Yuanhou Town, Chishui City, Guizhou Province, China. Taxonomic identification was performed by Dr. Li Zhi (Professor of Plant Systematics, School of Forestry, Guizhou University). Voucher specimens (accession number: LZ-20240106) were deposited in the Guizhou University Forestry Herbarium (GZAC). Fresh, healthy leaves from disease-free individuals were selected for genomic analysis, snap-frozen in liquid nitrogen, and stored at − 80 °C until processing. High-quality genomic DNA was extracted from leaf tissues using the Plant Genomic DNA Kit (DP305, Tiangen Biotech, Beijing, China). Sequencing was conducted on complementary platforms to ensure comprehensive coverage: short-read sequencing via the Illumina NovaSeq 6000 system (San Diego, CA, USA) and long-read sequencing using the Oxford Nanopore PromethION platform (Oxford, UK).

### Genome assembly and annotation

Long-read PromethION data underwent initial alignment to reference gene sequences using Minimap2 (v2.1) [30] for mitogenome reconstruction, followed by error correction with Canu (v2.2) [31]. To enhance assembly accuracy, second-generation sequencing (Illumina) reads were mapped to the corrected long-read sequences using Bowtie2 (v2.3.5.1) [32]. A hybrid assembly strategy was then implemented with Unicycler (v0.4.8) [33], integrating both short-read and corrected long-read datasets under default parameters. The resulting assembly was visualized and refined through manual inspection using Bandage (v0.8.1) [34] to resolve topological ambiguities.For mitochondrial genome annotation, GeSeq [35] was employed with the *Camellia sinensis* mitochondrial genome (GenBank: PP212896) as a reference. The final annotation was manually curated to conform to a circular genome model. Chloroplast genome assembly utilized the same specimen’s Illumina data processed through GetOrganelle (v1.7.7.0) [36], followed by annotation with the Plastid Genome Annotator (PGA) [37]. Genome maps were generated using OGDRAW (v1.3.1) [38] to illustrate structural features.

### Validation of mitochondrial genome repeat-mediated recombination in *Camellia luteoflora*

To elucidate recombination patterns in intra- and inter-loop structures, we implemented a multi-step analytical workflow. First, circular sequences assembled by Unicycler were subjected to pairwise alignment using BLASTN v2.12.0 (parameters: e-value ≤ 1e-5), with subsequent filtration retaining only those alignments spanning ≤ 100 bp to exclude low-complexity regions [39]. For long-read validation, we imposed stringent mapping criteria requiring ≥ 500 bp flanking sequence coverage on both sides of repetitive elements to ensure reliable resolution of repeat structures. Next-generation sequencing data from three distinct platforms were systematically aligned against the assembly using minimap2 v2.28 (preset: -x map-ont), followed by iterative realignment and interactive visualization of consensus regions through the same software pipeline. This multi-platform validation strategy enhanced the robustness of structural determination particularly in complex repetitive regions.

### *Camellia luteoflora* mitochondrial genome repeat sequence analysis

To characterize repetitive elements in the mitochondrial genome of *C. luteoflora*, we conducted systematic analyses through two computational approaches. Firstly, microsatellite identification was performed using MISA (https://webblast.ipk-gatersleben.de/misa/) [40] with optimized detection thresholds: mononucleotide repeats required ≥ 10 iterations, dinucleotides ≥ 5 iterations, trinucleotides ≥ 4 iterations, while tetra-, penta-, and hexanucleotide motifs required ≥ 3 iterations. These thresholds were established to ensure biological relevance while minimizing false positives from random sequence variations. Subsequently, complex repeat architecture was investigated using REPuter (https://bibiserv.cebitec.uni-bielefeld.de/reputer) [41] with stringent parameters: an edit distance ≤ 3 and minimum repeat length ≥ 300 bp for detecting four types of sequence duplications - direct, inverted, palindromic, and complementary repeats. The dual analytical framework enabled comprehensive detection of both short tandem repeats and large-scale structural duplications, providing critical insights into the organization and evolutionary dynamics of this mitochondrial genome.

### *Camellia luteoflora* mitochondrial genome codon preference analysis

The mitochondrial genome of *C. luteoflora* underwent systematic nucleotide composition analysis, with annotated protein-coding genes (PCGs) being computationally extracted for downstream characterization. Codon usage bias was quantitatively assessed using CodonW v1.4.2 through three-dimensional metrics: (1) whole-genome base distribution patterns, (2) relative synonymous codon usage (RSCU) calculations, and (3) amino acid-specific codon preference profiling. The RSCU metric, defined as the ratio between observed codon frequency and its theoretical expectation under neutral evolution (RSCU = 1 indicates no bias), revealed distinct translational selection pressures. Codons with RSCU > 1 demonstrated preferential utilization, where magnitude positively correlated with selection intensity [41]. Notably, extreme RSCU values (> 1.6) were interpreted as molecular signatures of evolutionary optimization for translation efficiency or tRNA abundance adaptation.

### Prediction of RNA editing sites

In order to obtain the RNA editing information in the mitochondrial gene sequences of *C. luteoflora*, we utilized the PREPACT v3.0 software (http://www.prepact.de/prepact-main.php) to perform the calculations [42]. According to all the RNA editing sites of *C. luteoflora*, the RNA editing occurring at the first, second, and third codons was counted separately; combined with the amino acid changes, we analyzed the changes in hydrophilicity and hydrophobicity of amino acids induced by RNA editing and the changes in the start codon and the stop codon induced by RNA editing.

### Ka/Ks and nucleotide diversity analysis of the mitochondrial genome of *Camellia luteoflora*

To elucidate the evolutionary trajectory of *Camellia sinensis* mitochondrial genome, we implemented a three-tiered comparative framework with six congeneric species (C. tianeensis, C. fangchengensis, C. chekiangoleosa, C. sinensis cv. Rougui, C. sinensis var. pubilimba, and C. sinensis reference genome). Firstly, core-gene phylogeny was reconstructed using MEGA11 (algorithm: Neighbor-Joining, bootstrap = 1000) through whole mitochondrial genome alignment, establishing the topological relationships among taxa. Secondly, evolutionary constraint metrics were calculated via DnaSP v6.12.03: (1) Non-synonymous/synonymous substitution ratios (Ka/Ks) with sliding window analysis (window = 150 bp, step = 30 bp) to detect selection signatures, and (2) Nucleotide diversity (π) calculations (window = 1000 bp) identifying conserved versus hypervariable genomic regions. Thirdly, branch-specific selection patterns were decoded using site-model comparisons (dN/dS > 1 indicating positive selection).The integrated analysis revealed three evolutionary hotspots (nad4L, cob, rps12) showing strong purifying selection (Ka/Ks = 0.12–0.35), while cox1 exhibited elevated π values (0.027 ± 0.004) suggesting adaptive diversification. Notably, C. sinensis cv. Rougui displayed unique selection signatures in atp6 (Ka/Ks = 1.18, *p* < 0.05), potentially linked to cultivar-specific mitochondrial-nuclear coevolution. These computational insights delineate how selection pressures sculpt mitochondrial genome architecture during *Camellia* speciation and domestication processes [43].

### Fragments shared between mitotic and chloroplast genomes and phylogenetic tree analysis

The assembled *C. luteoflora* chloroplast genome and mitochondrial genome sequences were used to identify homologous fragments between the mitochondrial and chloroplast genomes using BLAST v2.9.0 [44]available on NCBI, with the screening criteria of ≥ 70% match, E-value ≤ 1e-5, and ≥ 30 bp in length, and the screened sequence fragments were visualized using Circos v0.65 [44]. The mitochondrial genomes downloaded from NCBI were from 27 species with *Ginkgo biloba* Linn as the outgroup. Gene sequences of 33 conserved genes from each species were identified and extracted using PhyloSuite v1.2.1 [45]. Conserved gene sequences were aligned using MAFFT v7.450 [46], and aligned sequences were concatenated for phylogenetic tree construction. The aligned sequences were concatenated and trimmed using trimAl (v1.4). Subsequently, model prediction was conducted with jmodeltest(v2.1.10), identifying the GTR model. The maximum likelihood phylogenetic tree was then constructed using RAxML (v8.2.10) with the GTRGAMMA model and a bootstrap value set to 1000 [47]. The chloroplast genome was selected for 28 species for whole genome sequence construction of the phylogenetic tree in the same way as for the mitochondrial genome. Finally, we used the iTOL v6 (https://itol.embl.de/) online website to visualize the phylogenetic tree [48].

## Results

### Assembly and annotation of the *Camellia luteoflora* mitochondrial genome

**Fig. 1:**
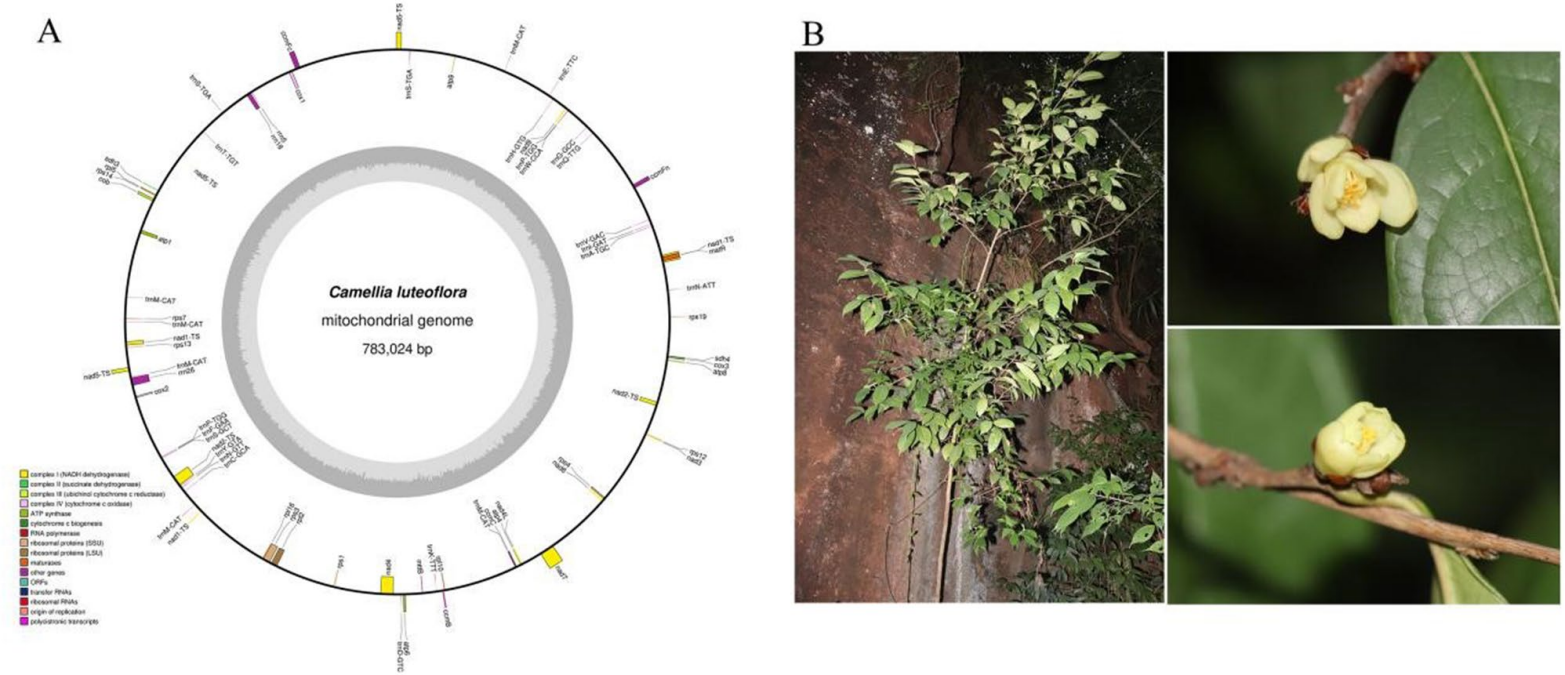
Mitochondrial genome assembly results of *Camellia luteoflora.* (**A**: Mitochondrial genome map; **B**: Field photo of *Camellia luteoflora*; The genes located in the upper region of linear molecules and within the interior of circular molecules represent genes transcribed in a clockwise direction, while thegenes in the lower region of linear molecules and the genes on the outside of circular molecules represent genes transcribed in a counterclockwise direction. Genes with different functions were depicted using different colors.)

*C. luteoflora* mitochondrial genome was assembled using both long and short sequence assembly strategies. For both long and short reads, we obtained about 6.58 Gb of nanopore high-quality reads with a readsN50 of 2000 bp and a total mass of 5.17 Gb. The short reads were assembled ab initio into a unitary map, and the contiguous-repetitive-contiguous regions were disassembled by the long reads. Finally, we obtained a single cyclic molecule with an average long-read coverage depth of 158.4×. The initial assembly map presented a complex conformation due to the presence of a large number of repetitive sequences. However, the utilization of ONT long read segments enabled us to represent the mitochondrial genome as a single circular molecule of 78,3024 bp. The GC content of the mitochondrial genome was 44.63%, which was significantly higher than the GC content of the chloroplast genome of the same species (39.03%) (Fig. 1).

The mitochondrial genome includes a total of 40 protein-coding genes, 27 tRNA genes and three rRNA genes (Table 1). The coding genes included five ATP synthase genes (*atp*1, *atp*4, *atp*6, *atp*8, and *atp*9), nine NADH dehydrogenase genes (*nad*1, *nad2*, *nad*3, *nad*4, *nad*4L, *nad*5, *nad*6, *nad*7, and *nad*9), four cytochrome c biogenesis genes (*ccm*B, *ccm*C, *ccm*FC and *ccm*FN), three cytochrome c oxidase genes (*cox*1, *cox*2, and *cox*3), one maturases gene (*mat*R), one membrane transporter protein gene (*mtt*B), and one ubiquitin cytochrome c reductase gene (*co*b). The variant genes include four ribosomal protein large subunits (*rpl*10, *rpl*16, *rpl*2, and *rpl*5), nine ribosomal protein small subunits (*rps*1, *rps*3, *rps*4, *rps*7, *rps*12, *rps*13, *rps*14, and two *rps*19 (one of which is a pseudogene)), and three succinate dehydrogenases (two *sdh*3 (one of which is a pseudogene) and sdh4). 3 *r*RNAs (*rrn*18, *rrn*26, and *rrn*5) and 27 *t*RNAs. Of these, the *nad*1, *nad*2, *nad*5, and *nad*7 genes contain 4 introns; the nad4 gene has 2 introns, and *rpl*2, *rps*3, *rps*1, and *ccm*FC have 1 intron. *trn*M-CAT and *trn*P-TGG genes are multicopy genes. *trn*A-TGC, *trn*I-GAT, *trn*S-TGA, and *trn*T-TGT genes contain 1 intron.

In addition, we obtained the complete chloroplast genome, which is 157,172 bp in length (Figure S1, Table S1). It contains a large single-copy (LSC) region of 86,725 bp, a small-size copy (SSC) region of 18,293 bp, and two inverted repeat regions (IRs) of 26,077 bp each. 131 genes were identified, including 87 PCGs, 8 rRNAs, and 36 tRNA genes.

**Table 1:** Results of mitochondrial genome annotation in C*amellia luteoflora*

Group of genes	Gene name
ATP synthase	*atp*1, *atp*4, *atp*6, *atp*8, *atp*9
Cytohrome c biogenesis	*ccm*B, *ccm*C, *ccm*Fc*, *ccm*Fn
Ubichinol cytochrome c reductase	*co*b
Cytochrome coxidase	*cox*1, *cox*2, *cox*3,
Maturases	*mat*R
Transport membrance protein	*mtt*B
NADH dehydrogenase	*nad*1****, *nad*2****, *nad*3, *nad*4**, *nad*4L, *nad*5****, *nad*6, *nad*7****, *nad*9
Ribosomal proteins (LSU)	*rpl*10, *rpl*16, *rpl*2*, *rpl*5
Ribosomal proteins (SSU)	#*rps*19, *rps*1*, *rps*12, *rps*13, *rps*14, *rps*19, *rps*3*, *rps*4, *rps*7
Succinate dehydrogenase	#*sdh*3, *sdh*3, *sdh*4
Ribosomal RNAs	*rrn*18, *rrn*26, *rrn*5
Transfer RNAs	*trn*A-TGC*, *trn*C-GCA, *trn*D-GTC, *trn*E-TTC, *trn*F-GAA, *trn*G-GCC, *trn*H-GTG, *trn*I-GAT*, *trn*K-TTT, *trn*M-CAT(6), *trn*N-ATT, *trn*N-GTT, *trn*P-TGG(2), *trn*Q-TTG, *trn*S-GCT, *trn*S-TGA, *trn*S-TGA*, *trn*T-TGT*, *trn*V-GAC, *trn*W-CCA, *trn*Y-GTA

Note: The * symbol indicate the number of introns; The # symbol represent pseudo genes; Numbers after gene names are the Number of copies of multi-copy genes

### Different configurations of mitochondrial genomes of *Camellia luteoflora*

**Fig. 2:**
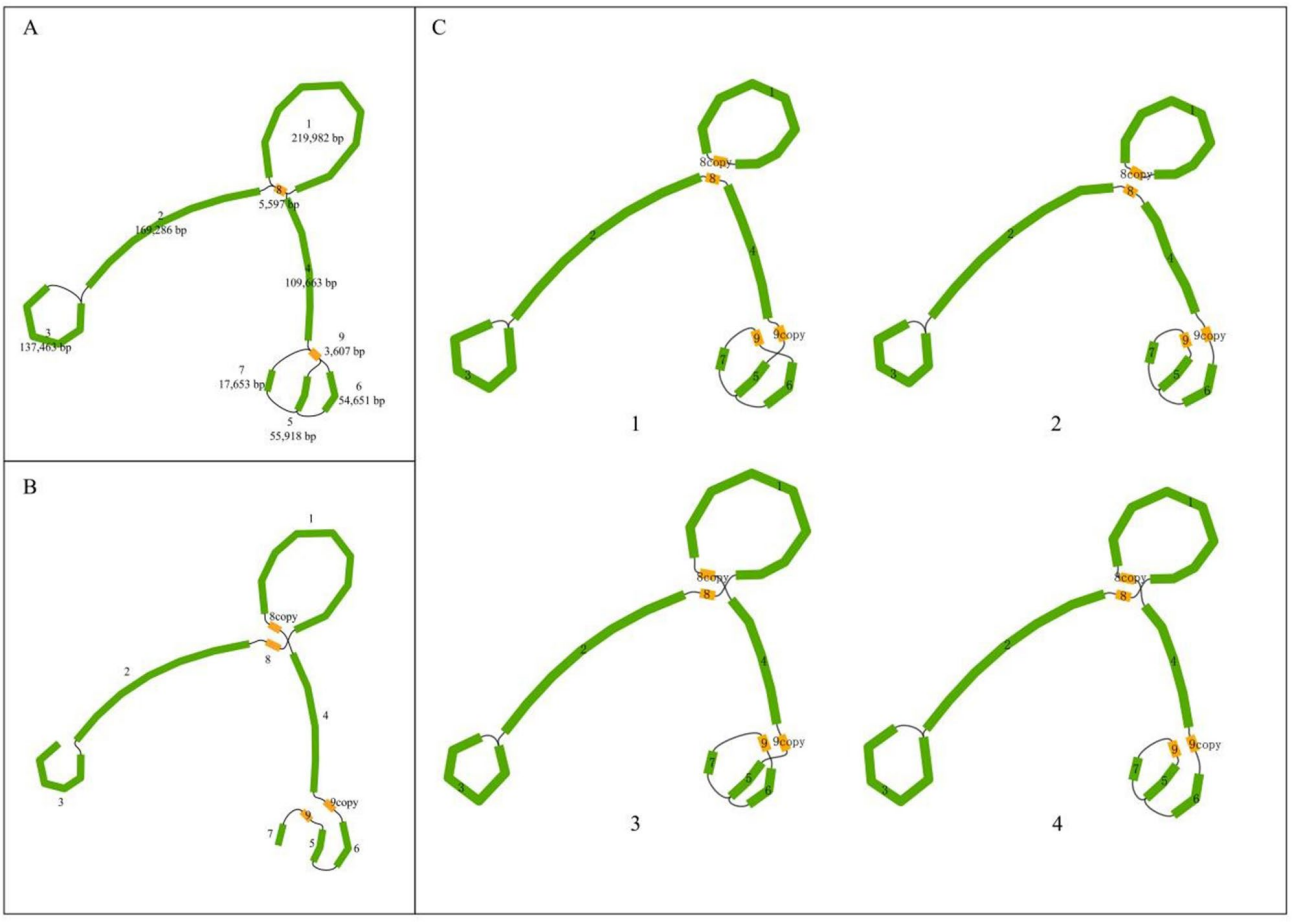
*Camellia luteoflora* recombination mediated by repetitive sequences. (The yellow rectangles indicate repetitive sequences at branch points. **A**: the initial structure of the genome is branching; **B**: the complete linear sequence of the genome branching unraveling; **C**: the four substructures arising from two pairs of repetitive sequences in the genome.)

**Fig. 3:**
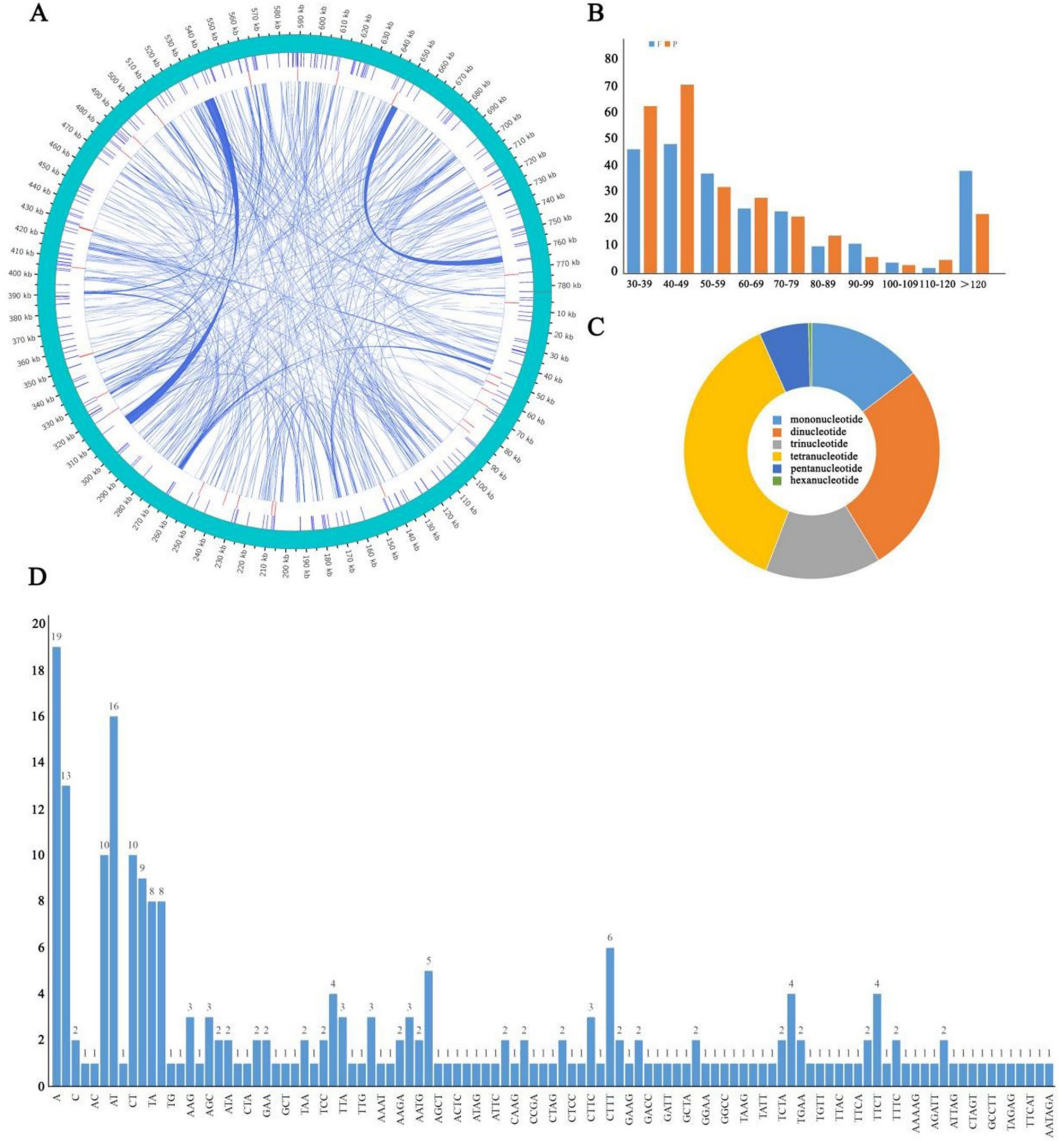
Repetitive sequences of *Camellia luteoflora* mitochondrial genome. (**A**: Distribution of repetitive sequences; **B**: length of forward repetitive sequence and palindromic sequence; **C**: proportion of six SSR types; **D**: number of SSR repetitive sequence types)

**Fig. 4:**
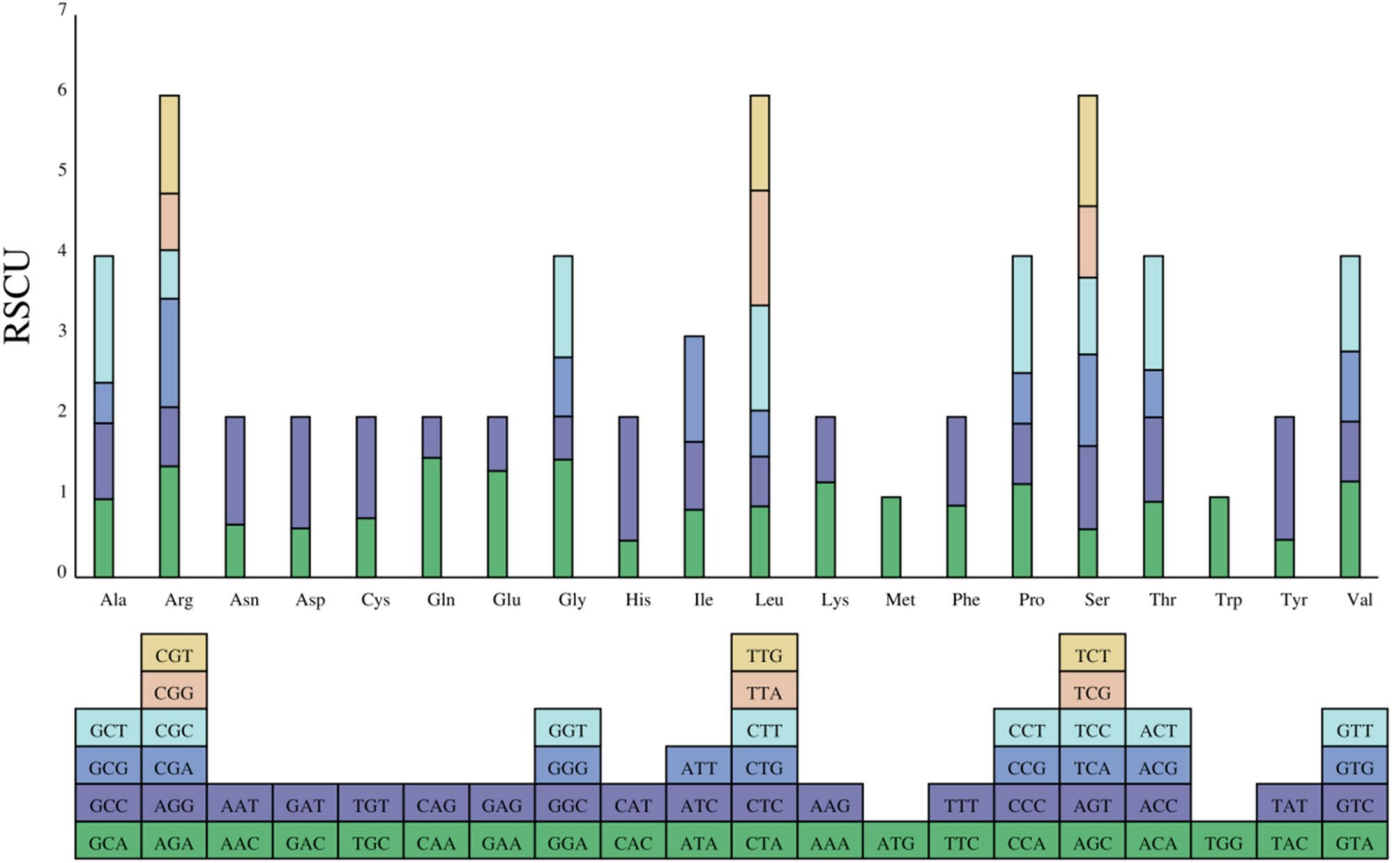
Use of relative synonymous codons in the mitochondrial genomes of *Camellia luteoflora*

Repeat sequences are frequently involved in homologous recombination, and in Fig. 2.Two of the 8 and 9 repeat sequences were identified as mediating recombination of consecutive sequences on the boundary. For repeat sequence 8, the sequence configuration in the assembled mitochondrial genome sequence was 1→8→1, 2→8→4 (Fig. 2C-1, Figs. 2C and 3). After recombination, the sequence configuration changed to 1→8→4, 2→8→1. Similarly, the sequence configuration of repeat sequence 8 was 7→9→5, 4→9→6 in the assembled mitochondrial genome sequence (Figs. 2C and C, 3 and 4). After recombination, the sequence configuration changed to 7→9→6, 4→9→5. The results illustrate the complexity of the mitochondrial genome structure of * C. luteoflora *(Fig. 2C).

In order to verify the existence of different configurations of the repeated sequences 8 and 9, we performed the sequences to verify the sequences 1→8→2→4 and 4→5→6→7→9, respectively. The results show that different combinations are supported across the repeated sequences, verifying the existence of different configurations of the repeated sequences. By mapping Nanopore longreads to sequences with different conformations associated with the repeat sequences, the study confirmed that intramolecular repeat sequences can mediate recombination (Figure S2-3). This phenomenon suggests that recombination mediated by specific homologous fragment pairs in the mitochondrial genome can influence the recombination patterns of different homologous fragment pairs, thereby enriching DNA substructures with different conformations.

### Repeat sequence analysis

In the *C. luteoflora* mitochondrial genome, two dispersed repeat sequence types, forward and palindromic, were identified, and a total of 506 dispersed repeats with lengths greater than or equal to 30 bp were obtained. Meanwhile, no forward and complementary repeat types were detected. These dispersed repeats were widely distributed throughout the intergenic region of the genome and were visualized by the Circos software package (Fig. 3A, Table S2). 506 repetitive sequences with a total length of 24,149 bp accounted for 3.08% of the total mitochondrial genome length. Among these repetitive sequences, there were 243 F repeats and 263 P repeats, and repeats of 40 to 49 bp were the most common (Figure. 3B, Table S3). A total of 240 SSRs were identified in the mitochondrial genome, and tetranucleotide repeats dominated with 37.50% (90) of the total number, followed by dinucleotide repeats (64), mononucleotide repeats and trinucleotide repeats (both 35), pentanucleotide repeats (15), and hexanucleotide repeats (1). Among the single nucleotide SSRs, the highest percentage of A repeats (54.29%) and among the dinucleotide repeats, the highest percentage of AT base repeats (25.00%) were found (Figure. 3 C, 3D; Table S4,5).

### *Camellia luteoflora* mitochondrial genome codon preference analysis

In the *C. luteoflora* mitochondrial genome, we found that the utilization rates of the termination codons UAA, UGA, and UAG were 50.00%, 31.58%, and 18.42%, respectively, while the termination codon TAG was not used. Relative synonymous codon usage (RSCU) can eliminate the effect of amino acid composition on codon usage and directly reflect the differences in codon usage patterns. The value of RSCU is equal to 1, which indicates an unbiased selection of codon usage.The value of RSCU is greater than 1, which means the frequency of usage is higher; the value of RSCU is less than 1, which means the frequency of usage is lower. is lower. The analysis of the RSCU method showed that there were two codons with RSCU equal to 1, which were AUG of Met and UGG of Trp; GCU codon encoding alanine (Ala) had the highest frequency, with an average RSCU value of 1.5766; and there were 29 codons with RSCU greater than 1, and most of them ended with A or U (Fig. 4, Table S6).

### Mitochondrial plastid DNA (MTPTs) in the mitochondrial genome of *Camellia luteoflora*

**Fig. 5:**
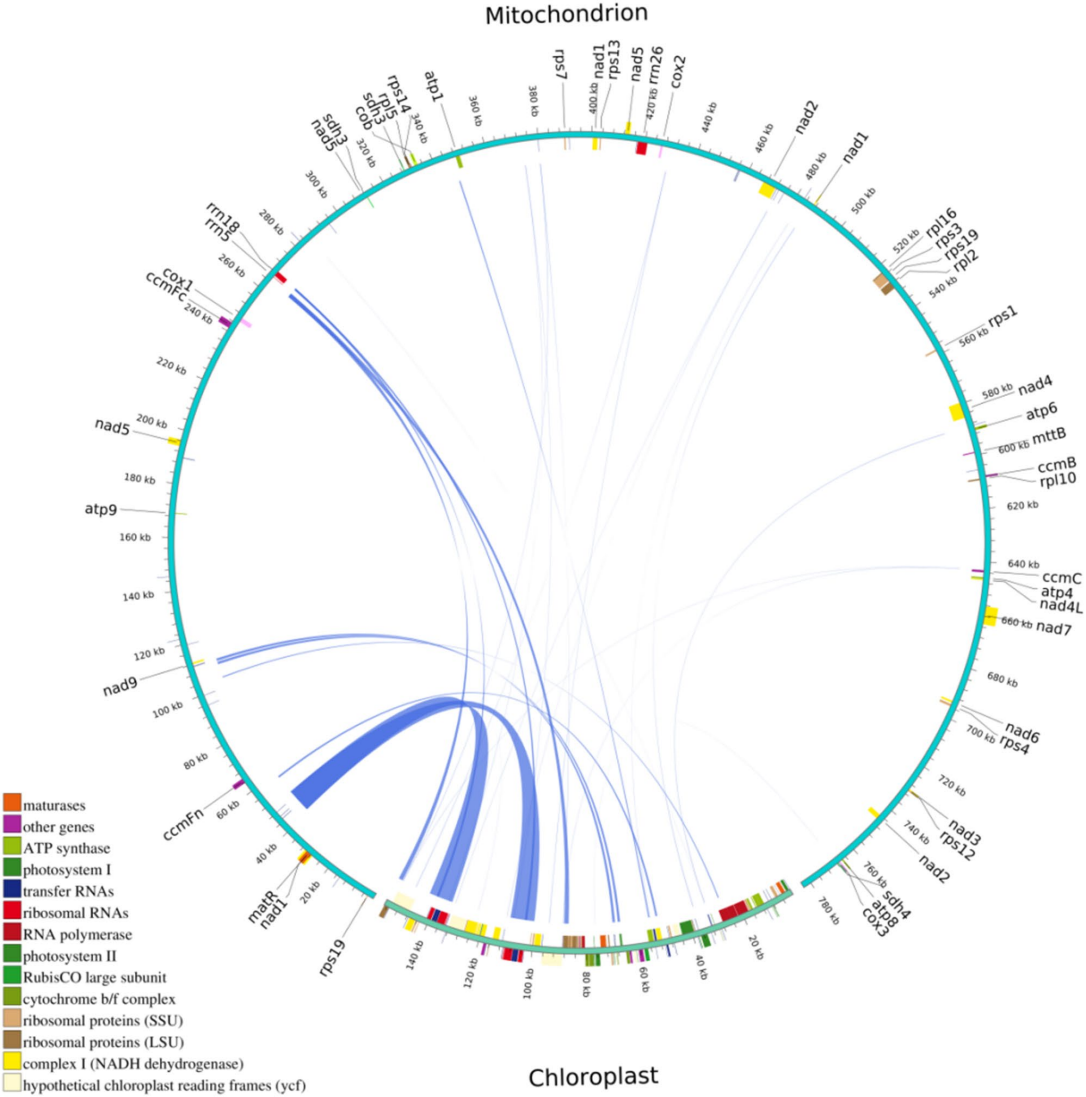
Fragments of *Camellia luteoflora* converted from chloroplasts to mitochondria

Intracellular transfer of genetic material has been a common phenomenon in mitochondrial genomes during the evolution of higher plants, and these sequence fragments originating from chloroplast organelles are relatively poorly conserved. The mitochondrial genome of *C. luteoflora* is approximately 4.9 times larger than the chloroplast genome (157,172 bp). The distribution of mitochondrial genes was relatively sparse compared to chloroplasts (Fig. 5). Based on the sequence similarity between the chloroplast and mitochondrial genomes, 19 chloroplast gene fragments were identified to be transferred to the mitochondrial genome in this study. The total length of these transferred fragments was 29,534 bp, representing 3.77% of the entire mitochondrial genome. Referring to these fragments as MTPTs, they represent sequence migration from chloroplasts to mitochondrial organelles (Fig. 5). Among these 19 homologous fragments, the mitochondrial genome consists of nine CDS regions, two rRNA genes, and eight tRNAs. The longest sequence of MTPT1 was 9572 bp, and the shortest sequence of MTPT19 was 32 bp (Table 2). It is noteworthy that the two rRNA genes and eight tRNA genes present in the chloroplast genome of *C. luteoflora* may have been lost or undergone pseudogene changes in the chloroplast genome.

**Table 2:** Transfer of *Camellia luteoflora* Chloroplast genome to mitochondrial genome gene fragments

No	Length	Identity	Mt-start	Mt-end	Cp-start	Cp-end	Type
1	9572	1.00	50,542	40,971	100,427	109,998	*trnV-GAC* tRNA
	9572	1.00	40,971	50,542	133,900	143,471	
2	1689	1.00	265,783	264,095	86,786	88,474	*rpl23* CDS
	1689	1.00	264,095	265,783	155,424	157,112	
3	1023	0.87	110,407	111,381	68,348	69,362	*trnW-CCA* tRNA
4	992	0.86	109,008	109,995	66,337	67,278	*psbJ* CDS
5	758	0.82	58,427	57,693	51,402	52,144	*ndhJ* CDS
6	505	0.87	346,892	347,383	54,578	55,064	*atpE* CDS
7	416	0.82	103,427	103,834	25,936	26,325	*rpoB* CDS
8	890	0.74	268,532	267,674	103,377	104,240	*rrn16* rRNA
	890	0.74	267,674	268,532	139,658	140,521	
9	147	0.96	485,957	486,103	36,687	36,833	*psbC* CDS
10	149	0.91	589,085	588,937	31,921	32,069	*trnD-GUC* tRNA
11	127	0.94	379,950	379,824	155,033	155,159	*trnI-CAU* tRNA
	127	0.94	379,824	379,950	88,739	88,865	
12	126	0.89	429,969	429,850	94,366	94,491	*ycf2* CDS
	126	0.89	429,850	429,969	149,407	149,532	
13	116	0.91	373,983	373,875	108,588	108,703	*rrn23* rRNA
	116	0.91	373,875	373,983	135,195	135,310	
14	84	0.96	473,229	473,146	132,470	132,552	*trnN-GUU* tRNA
	84	0.96	473,146	473,229	111,346	111,428	
15	77	0.94	482,871	482,947	54,264	54,340	*trnM-CAU* tRNA
16	76	0.93	643,141	643,066	155,033	155,108	*trnI-CAU* tRNA
	76	0.93	643,066	643,141	88,790	88,865	
17	41	1.00	49,911	49,871	123,002	123,042	*ndhA* CDS
18	34	0.97	768,270	768,237	76,283	76,316	*psbB* CDS
19	32	0.97	285,986	286,017	32,027	32,058	*trnD-GUC* tRNA

Note: Mt: mitochondrial genome; Cp: chloroplast genome

### RNA editing events in the mitochondrial genome of *Camellia luteoflora*

**Fig. 6:**
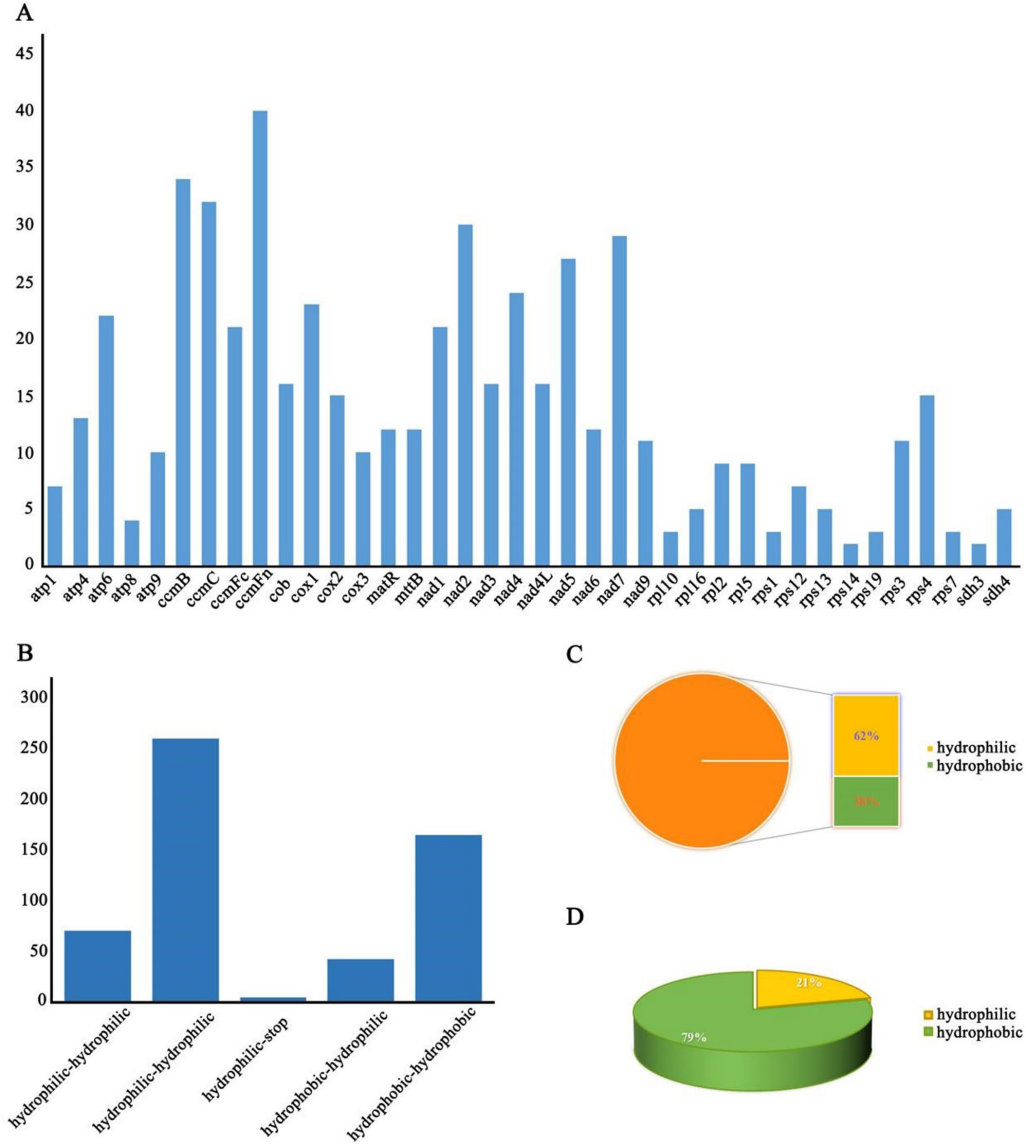
Prediction of RNA editing sites for PCGs in the mitochondrial genome of *Camellia luteoflora.* (**A**: number of RNA editing sites for each gene; **B**: type of amino acid conversion **C**: percentage of hydrophilic and hydrophobic amino acids before RNA editing; **D**: percentage of hydrophilic and hydrophobic amino acids after RNA editing.)

In this study, we revealed the characteristics of post-transcriptional modifications by predicting the RNA editing sites of all PCGs in the mitochondrial genome of *C. luteoflora*. The results showed that a total of 539 RNA editing events were identified, which were mainly characterized by base C to T (corresponding to base C to U in RNA) transitions (Table S7). The ccmFn gene was the most significant, with 40 editing sites identified, followed by the ccmB gene (34 editing sites). In addition, rps14 and sdh3 each had 2 RNA editing sites (Fig. 6A). The study further observed that the editing events were concentrated at the first and second base positions of the start codon. These RNA editing events resulted in amino acid changes such as histidine (His) to tyrosine (Tyr), arginine (Arg) to cysteine (Cys), threonine (His) to isoleucine (Iso), leucine (Leu) to phenylalanine (Phe), serine (Ser) to phenylalanine (Phe), arginine (Arg) to tryptophan (Try), etc. A total of five amino acid type shifts were also found, with the most being hydrophilic to hydrophobic conversions and the least being hydrophilic to stop conversions (Fig. 6B). The results showed that the ratio of hydrophilic and hydrophobic types before and after the amino acid changes was quite different, with the ratio changing from 1.63 to 0.27 before and after editing (Fig. 6C, D). It can be seen that many of the amino acid changes triggered by RNA editing introduce more hydrophobic amino acids into the protein structure, thereby altering the hydrophilic nature of the protein, which may play a key role in maintaining the regulation of mitochondrial gene expression.

### Selection pressure on the mitochondrial genome of *Camellia luteoflora* plants and its nucleotide diversity analysis

**Fig. 7:**
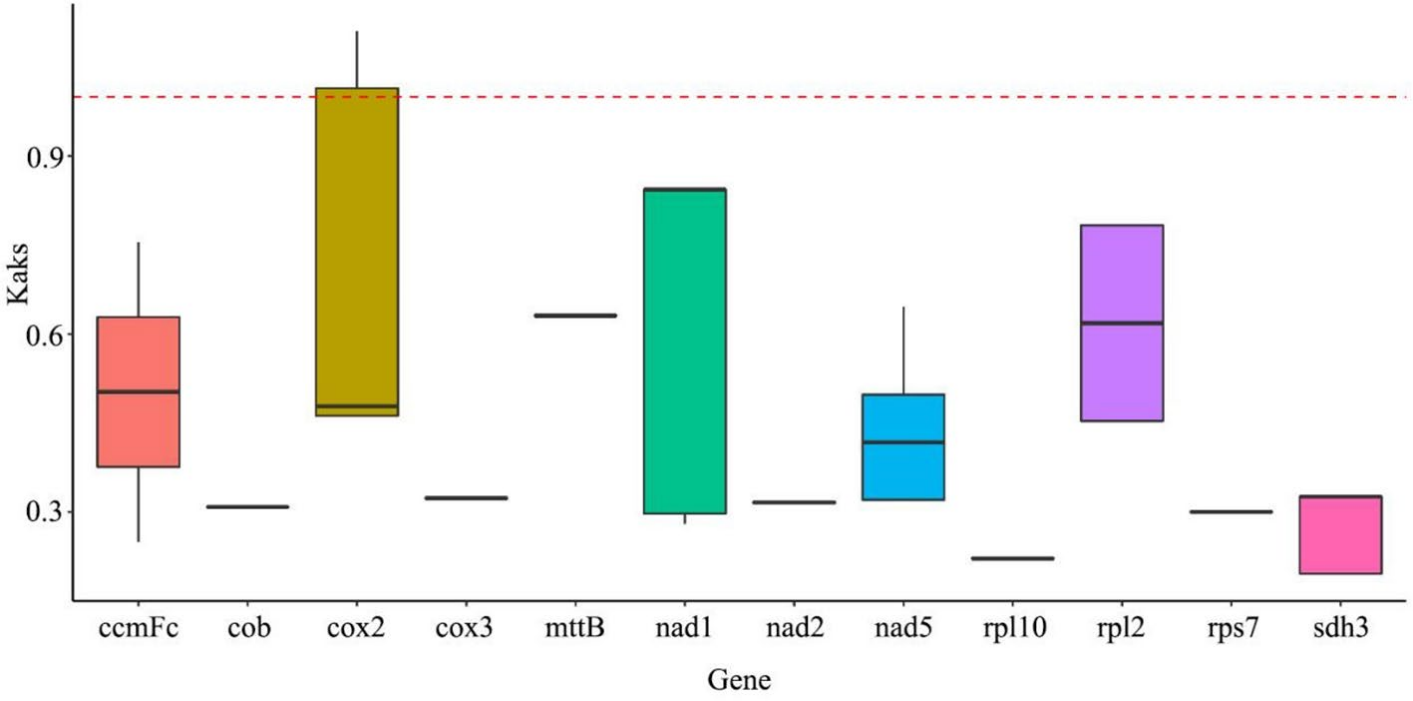
Box plots of Ka/Ks ratios of *Camellia luteoflora* with six plants

Ka/Ks (also known as dN/dS) denotes the ratio of the rate of nonsynonymous substitutions (Ka) to the rate of synonymous substitutions (Ks) and is used to measure the selective pressure on proteins during the evolution of different species. When Ka/Ks > 1, the gene is under positive selection. When Ka/Ks = 1, genes undergo neutral evolution. When Ka/Ks < 1, genes are subject to negative or purifying selection. In order to assess the selection pressure on *C. luteoflora* and its relatives, PCGs, the selection pressure on seven *Camellia* L. was analyzed. The results showed (Fig. 7, Table S8) that the average Ka/Ks value of the 12 genes was 0.45. Only the *cox*2 gene had a Ka/Ks value greater than 1, indicating that it was subject to strong positive selection. The remaining 11 genes had Ka/Ks values less than 1. This phenomenon reveals that these genes have been subject to strong negative selection during evolution and have relatively stable protein functions. These 12 protein-coding genes of *C. luteoflora* mitochondria showed consistent conserved properties at the molecular evolutionary level with their counterparts in the six selected plants and were less susceptible to interspecies genetic variation. This analysis is of great scientific value for understanding the molecular evolutionary pathways of *C. luteoflora* and other species, the conservatism and innovativeness of gene functions, and the genetic basis of adaptive differences among species.

**Fig. 8:**
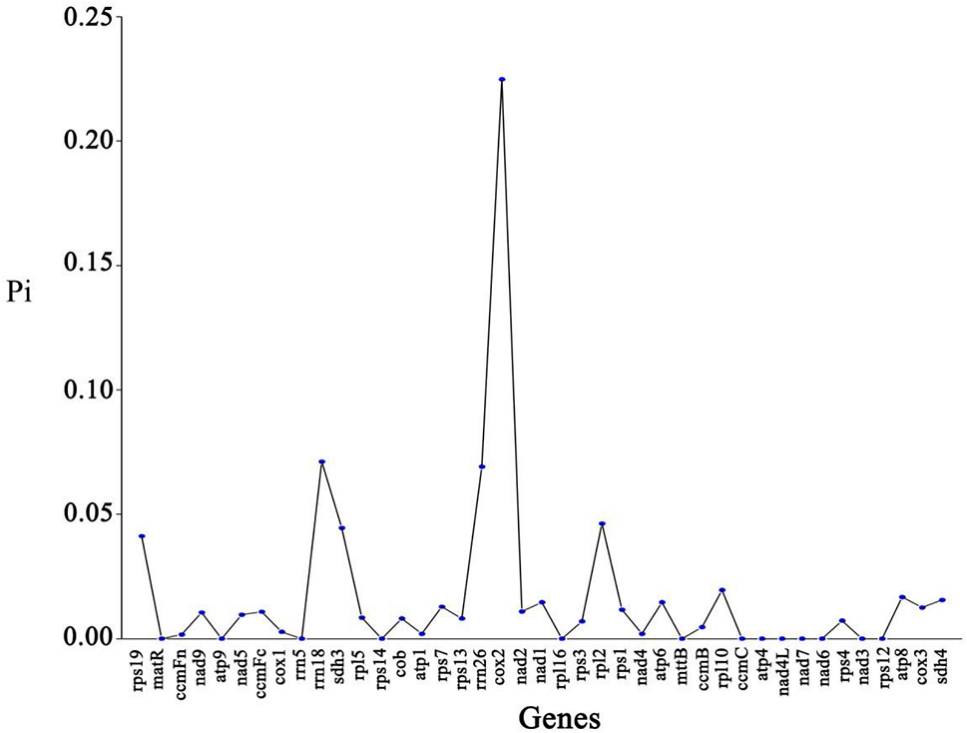
Nucleotide diversity values of *Camellia luteoflora* with six species

Nucleotide diversity (Pi) can be used to assess genetic differences in nucleotide sequences between species and populations and to select regions of high variability as potential molecular markers for populations. Pi analysis of organelle genes was performed on seven *Camellia* L. The results showed (Fig. 8, Table S9) that the mitochondrial gene with the highest variability was *cox*2 (Pi = 0.02248), followed by *rrn*18 (Pi = 0.00711) and *rrn*26 (Pi = 0.00691). In mitochondrial PCG, the Pi values of all genes ranged from 0 to 0.00711, indicating that the nucleotide sequences of *C. luteoflora* mitochondrial genes are highly conserved.

### Construction of phylogenetic tree of *Camellia luteoflora* mitochondrial genome

**Fig. 9:**
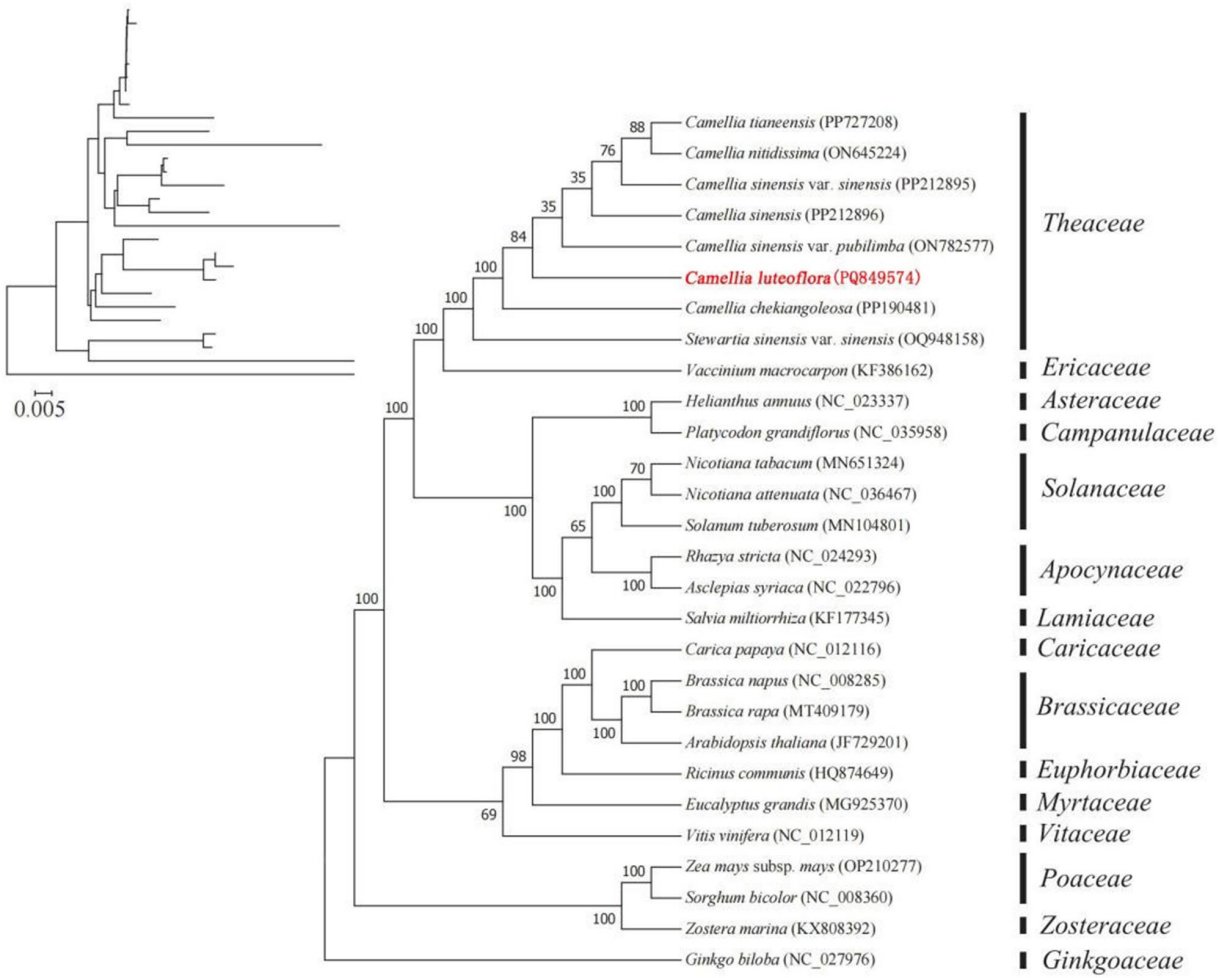
Phylogenetic relationships of *Camellia luteoflora* mitochondrial genomes. ( phylogenetic tree constructed on the basis of 28 protein coding genes)

**Fig. 10:**
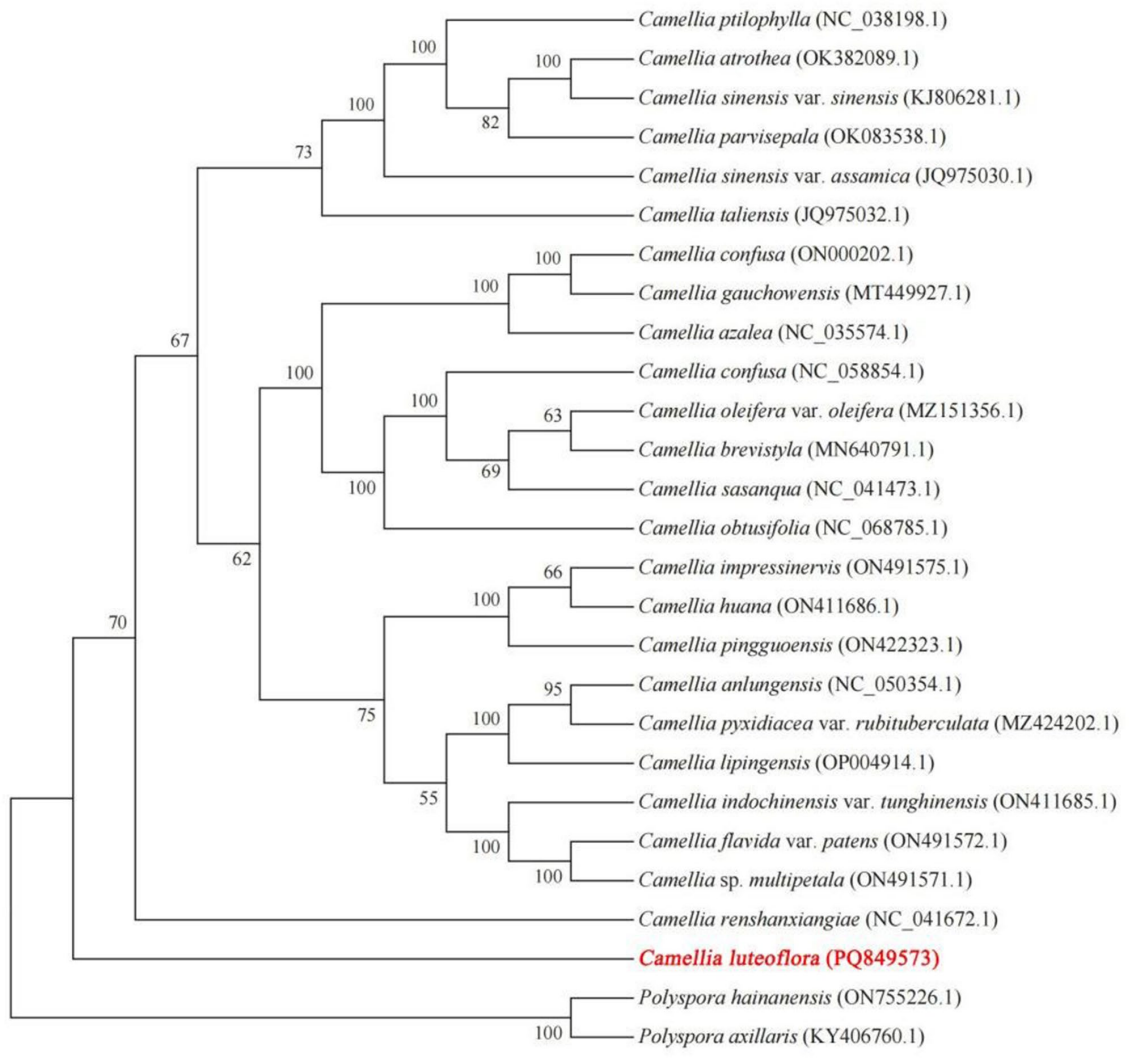
Phylogenetic relationships of *Camellia luteoflora* chloroplast genomes. (phylogenetic tree constructed on the chloroplast genome)

To determine the phylogenetic position of *C. luteoflora*, we constructed a phylogenetic tree for green with protein-coding genes from the mitochondrial genome and the whole chloroplast genome. The phylogenetic tree was constructed based on 33 shared coding genes of the mitochondrial genomes of 28 species using *Ginkgo biloba* as an outgroup. The results showed (Fig. 9) that the phylogenetic tree strongly supported the delineation of dicots from monocots and the separation of angiosperms from gymnosperms, and that the phylogenetic relationships of these species were consistent with traditional classification. Meanwhile, the closest relative to *C. luteoflora* in the genus *Camellia* was *Camellia sinensis* var. *sinensis*, followed by *Camellia sinensis*, and the most distant species was *Stewartia sinensis* var. sinensis. using *Polyspora axillaris* and *Polyspora hainanensis* as outgroups, the phylogenetic tree constructed based on the whole chloroplast genes of 28 species (Fig. 10) showed that sect. *luteoflora* was in a separate position in *Camellia* L. and was more closely related to the sect. *Camellia*, while sect. *Thea* was more distantly related.

## Discussion

With the continuous advancement of sequencing technologies, research on chloroplast genome sequences of higher plants has accumulated substantial achievements. In contrast, studies on plant mitochondrial genomes remain relatively limited, though this field has now emerged as a burgeoning research focus [49]. The primary challenges in plant mitochondrial genome studies stem from their multifaceted complexity: intricate genomic composition, structural diversity, abundant recombinogenic repetitive sequences, and dynamic conformational changes. The interaction mechanisms between mitochondrial and nuclear genomes, coupled with their central roles in energy metabolism and biosynthesis, render functional and regulatory investigations particularly challenging. Therefore, deciphering mitochondrial structure and function is crucial for unraveling key physiological phenomena and evolutionary patterns in biology, yet requires overcoming multiple technical obstacles.In recent years, the increasing number of assembled plant mitochondrial genomes [50, 51] has enabled comprehensive comparative genomic studies. Significant interspecies variations have been observed in mitochondrial genome characteristics including total base length, gene composition, gene arrangement order, GC content, and overall architecture [52, 53]. Structurally, mitochondrial genomes typically exhibit complex circular or linear configurations. While most plants possess single circular genomes, some species such as Salvia japonica and Paphiopedilum micranthum demonstrate multipartite genome structures [54, 55].

In this study, we employed high-throughput sequencing to assemble the mitochondrial genome of *C. luteoflora*, which spans 587,847 bp. Through comprehensive annotation, we identified 42 protein-coding genes (PCGs), 3 rRNA-encoding genes, and 27 tRNA-encoding genes. The GC content of plant mitochondrial genomes has been recognized as an indicator of species adaptation [56], with documented values ranging from 23.9 to 50.5% in terrestrial plants [16]. Notably, the mitochondrial genome of *C. luteoflora* exhibited a GC content of 44.63%, significantly exceeding that of its chloroplast genome (39.03%).The mitochondrial genome architecture of *C. luteoflora* features multiple interconnected gene segments organized into a continuous, high-resolution network. These structurally integrated components are functionally critical for mitochondrial operations, potentially encoding essential proteins for cellular energy production. Further analysis revealed intricate organizational details including gene quantity, spatial arrangement patterns, and putative regulatory elements. These structural insights establish a foundation for elucidating mitochondrial gene functionality and associated expression regulation mechanisms.

Repetitive sequences serve as crucial elements for investigating genome architecture, gene expression regulation, phenotypic characteristics, molecular marker development, functional annotation, and evolutionary processes [57–59]. Compared to chloroplast and nuclear genomes, plant mitochondrial genomes demonstrate significantly slower evolutionary rates [60], making their molecular markers particularly valuable for enhancing species identification precision. In this study, we identified 506 repetitive sequences in the *C. luteoflora* mitochondrial genome, comprising 243 forward (F) and 263 palindromic (P) repeats. These repetitive elements suggest active molecular recombination events that likely drive dynamic structural rearrangements and conformational modifications during mitochondrial genome evolution. Additionally, 240 simple sequence repeats (SSRs) were detected across distinct genomic regions. The SSR profile exhibited tetranucleotide repeats as the predominant type (37.50%), followed by dinucleotide repeats (26.67%), a distribution pattern consistent with observations in most angiosperm mitochondrial genomes [41].

Codons serve as fundamental units in biological genetic expression, mediating protein synthesis, modulating gene expression, and facilitating genetic variation [61]. Organisms develop codon usage bias through selective pressures from environmental constraints and species-specific evolutionary trajectories [62], with these preferential synonymous codon selections critically shaping genomic characteristics.Our analysis reveals that the *C. luteoflora* mitochondrial genome exhibits pronounced A/T nucleotide enrichment, demonstrating strong preferential usage of codons terminating with A/U bases. Comparative studies with other plant mitochondrial genomes [41, 63–65] indicate that while A/U-ending codon preference represents a conserved feature across green plant lineages, the degree of bias displays both interclade variations and intragroup fluctuations. The observed A/U predominance in *C. luteoflora* provides supporting evidence for ancestral codon usage patterns in early-diverging land plants. Phylogenetic conservation of mitochondrial codon preferences likely originates from the monophyletic nature of plant mitochondria, whereas bias intensity variations may reflect terrestrial adaptation processes. Notably, the evolutionary transition from strong AT-bias toward gradual GC accumulation in derived lineages [65] potentially represents an adaptive strategy to mitigate UV-induced DNA damage in xeric environments.

Exogenous gene insertions in plant mitochondrial genomes predominantly localize to intergenic regions. The integration length of chloroplast-derived DNA sequences exhibits interspecific variation, typically constituting 1–12% of chloroplast genome content in angiosperms [40, 66, 67]. This transfer mechanism represents a primary driver of gene content divergence among plant mitochondrial genomes, underscoring the necessity of tracking gene migration patterns for evolutionary studies [68]. Chloroplast-to-mitochondria tRNA gene transfers constitute a widespread phenomenon in plants [69]. Our investigation identified eight chloroplast-origin tRNA genes in the *C. luteoflora* mitochondrial genome, potentially fulfilling functional compensation roles. Additionally, we detected numerous chloroplast-derived gene fragments containing chloroplast-specific functional elements, though their mitochondrial operational significance remains unconfirmed.

Post-transcriptional RNA editing modifies genetic information, resulting in mitochondrial protein products often deriving from partially edited transcripts. Hydrophilic amino acids critically influence protein folding processes, with reduced proportions correlating with enhanced structural stability [70]. In C. luteoflora, mitochondrial RNA editing predominantly converts hydrophilic to hydrophobic residues, consequently increasing overall protein hydrophobicity. Ka/Ks calculations provide critical insights for phylogenetic reconstruction and protein evolution analysis [71]. Our results demonstrate prevalent negative selection pressure on mitochondrial coding genes, aligning with established patterns [41]. Notably, *cox*2 exhibited Ka/Ks > 1, suggesting its pivotal evolutionary role in *Camellia* mitochondrial genome development.

Plant mitochondrial genomes exhibit frequent structural rearrangements and gene content flux, driven by mitochondrial-nuclear DNA transfers and suppressed mutation rates [72]. These evolutionary signatures offer unique phylogenetic markers [73], enabling species relationship reconstruction through mitochondrial gene homology analysis [74]. In this study, the phylogenetic relationships of *Camellia luteoflora* were constructed based on mitochondrial and chloroplast genomic information. The mitochondrial genome was able to strongly support the delineation of dicots from monocots and the separation of angiosperms from gymnosperms, and the species *Camellia sinensis* var. *sinensis*, which is closely related to *C. luteoflora*, was found. Meanwhile, phylogenetic analyses constructed from the chloroplast genome further confirmed the separate status of sect. *luteoflora* from *Camellia* L. sect. *Camellia* that are more closely related to it were found, but these still need to be further proved by biogeographical evidence.

## Conclusion

*C. luteoflora* is a plant with important economic and medicinal value, as well as a more specialized taxon of *Camellia* L. This study pioneers the assembly of *C. luteoflora* mitochondrial genome through integrated sequencing approaches and comprehensive bioinformatic analyses. These advancements enable systematic comparisons of organellar genome architectures while expanding investigative perspectives on mitochondrial-plastid DNA transfer mechanisms.Phylogenetic reconstruction based on mitochondrial and chloroplast genomic datasets corroborates the species’ evolutionary position within its taxonomic clade. The findings establish foundational references for understanding *C. luteoflora*’s genetic characteristics, molecular variation patterns, evolutionary origins, and systematic classification, while concurrently informing cultivation practices and resource utilization strategies.To refine phylogenetic resolution within *Camellia* L., future investigations should prioritize mitochondrial genome sequencing across broader taxonomic representatives.

## Electronic supplementary material

Below is the link to the electronic supplementary material.

Supplementary Material 1: Fig. S1: Chloroplast genome map of *Camellia luteoflora*;

Supplementary Material 2: Figure S2. Verification of repeat 8 mediated recombination coverage in *Camellia luteoflora*;

Supplementary Material 3: Figure S3. Verification of repeat 9 mediated recombination coverage in *Camellia luteoflora*;

Supplementary Material 4: Table S1. Characteristics of the chloroplast genome of *Camellia luteoflora*;

Supplementary Material 5: Table S2. Distribution of repetitive sequences;

Supplementary Material 6: Table S3. Length analysis of dispersed repetitive sequences;

Supplementary Material 7: Table S4. Proportion of six SSR types;

Supplementary Material 8: Table S5. Number of SSR repetitive sequence types;

Supplementary Material 9: Table S6. Use of relative synonymous codons in the mitochondrial genomes of *Camellia luteoflora*;

Supplementary Material 10: Table S7. Prediction of RNA editing sites for PCGs in the mitochondrial genome of *Camellia luteoflora*;

Supplementary Material 11: Table S8. KaKs ratios of *Camellia luteoflora* with six plants;

Supplementary Material 12: Table S9. Nucleotide diversity values of *Camellia luteoflora* with six species;

Supplementary Material 13: Table S10. Phylogenetic tree constructed on the basis of 28 Mitochondrial genome;

Supplementary Material 14: Table S11. Chloroplast genome sequence numbers of the 28 species used to construct the phylogenetic tree

## Data Availability

No datasets were generated or analysed during the current study.

## Abbreviations

PCGs: Protein-Coding Genes
mtDNA: Mitochondrial Genome
cpDNA: Chloroplast Genome
Ka/Ks: Non-Synonymous/Synonymous Mutation Ratio
RSCU: Relative Synonymous Codon Usage
MTPT: Mitochondrial Plastid DNA Sequence
tRNA: Transfer RNA
rRNA: Ribosomal RNA
SSR: Simple Sequence Repeat
Pi: Nucleotide Diversity
BS: Bootstrap Support Value
PP: Posterior Probabilities
PPR: Pentatricopeptide Repeat
