# Supplementary figures and images for: Mitochondrial genome assembly of the Chinese endemic species of Camellia luteoflora and revealing its repetitive sequence mediated recombination, codon preferences and MTPTs
Source: BMC Plant Biol. 2025 Apr 5;25:435. doi: 10.1186/s12870-025-06461-6 (PMC11971748; doi:10.1186/s12870-025-06461-6)

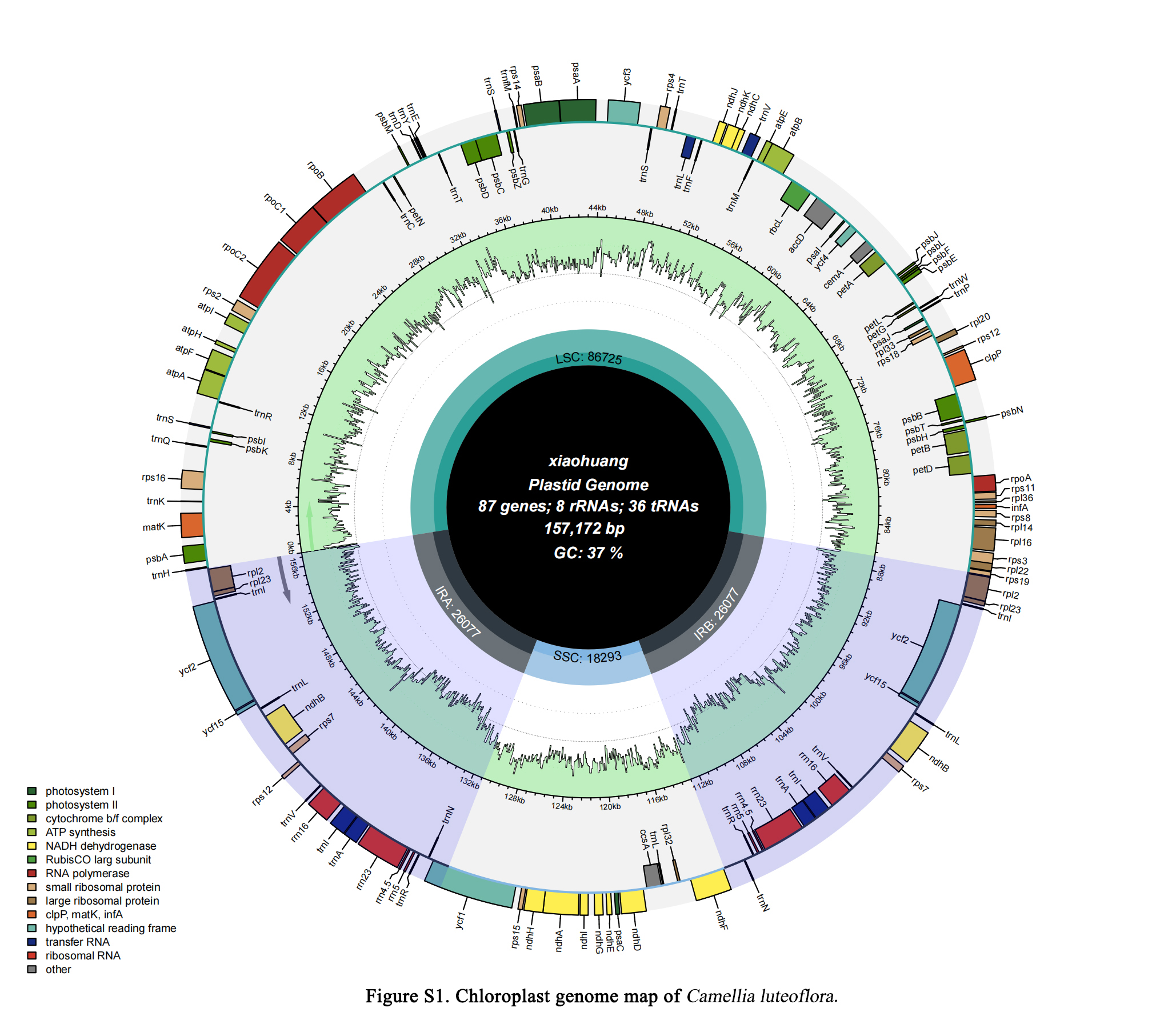

Supplement: Supplementary file 1 — Supplementary Material 1: Fig. S1: Chloroplast genome map of Camellia luteoflora; [file 12870_2025_6461_MOESM1_ESM.jpg]

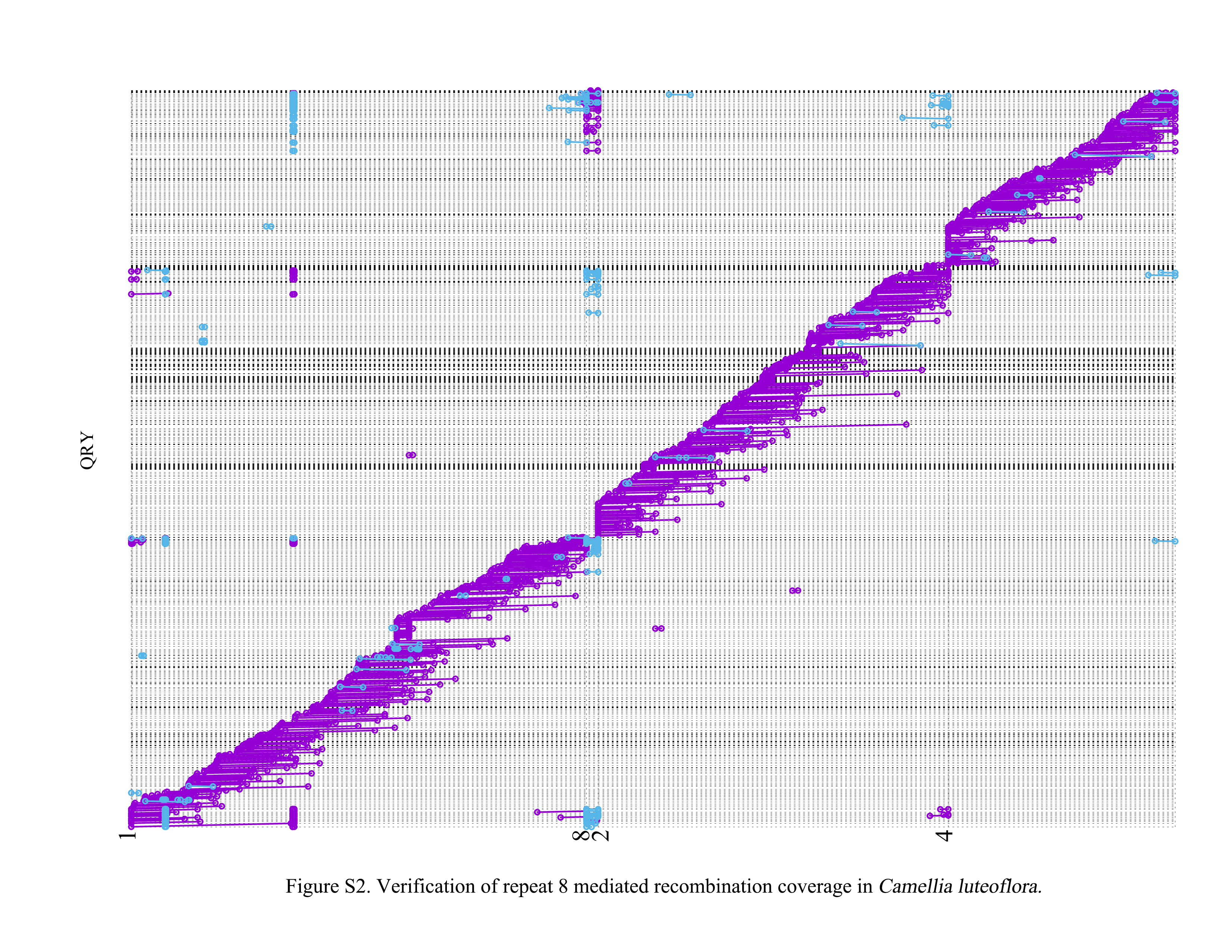

Supplement: Supplementary file 2 — Supplementary Material 2: Figure S2. Verification of repeat 8 mediated recombination coverage in Camellia luteoflora; [file 12870_2025_6461_MOESM2_ESM.jpg]

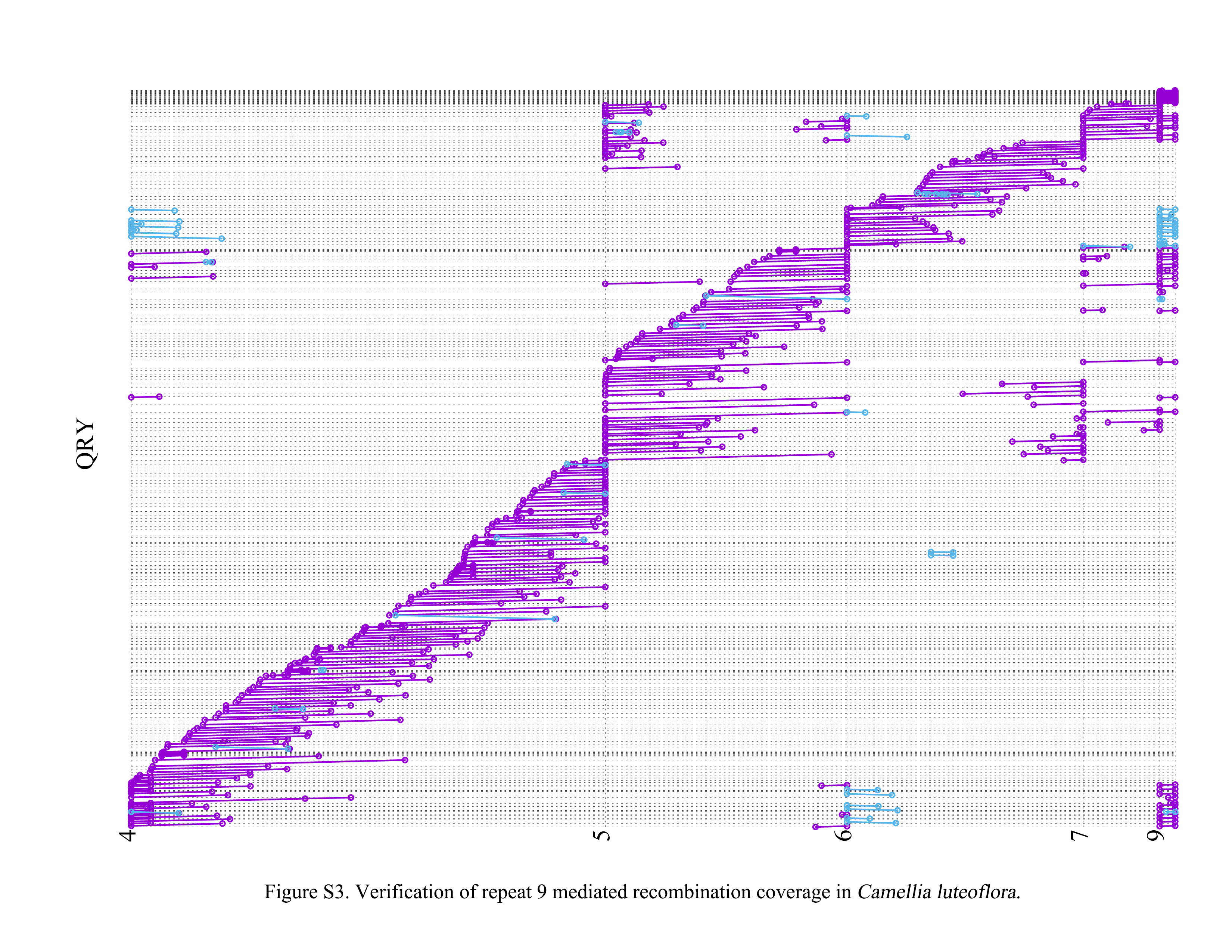

Supplement: Supplementary file 3 — Supplementary Material 3: Figure S3. Verification of repeat 9 mediated recombination coverage in Camellia luteoflora; [file 12870_2025_6461_MOESM3_ESM.jpg]
